# MR-guided microwave ablation of liver tumors: outcomes in local tumor control and determinants of treatment success

**DOI:** 10.1007/s00330-026-12725-y

**Published:** 2026-07-04

**Authors:** Vanessa F. Schmidt, Philipp Linden, Olaf Dietrich, Sinan Deniz, Daniel Puhr-Westerheide, Osman Öcal, Moritz L. Schnitzer, Florian Obereisenbuchner, Matthias Kassube, Mingming Wu, Luigi Nardone, Lars Grenacher, Florian Maier, Ricarda Seidensticker, Moritz Wildgruber, Jens Ricke, Matthias P. Fabritius, Max Seidensticker

**Affiliations:** 1 Department of Radiology, LMU University Hospital, LMU Munich, München, Germany; 2 Department of Diagnostic and Interventional Radiology, Heidelberg University Hospital, Heidelberg, Germany; 3 Department of Medicine, Institute of Radiology, University of Udine, Udine, Italy; 4 Conradia Radiology Munich, Munich, Germany; 5 Siemens Healthineers, Erlangen, Germany

**Keywords:** Magnetic resonance imaging, Image-guided therapy, Ablation techniques, Microwaves, Treatment outcome

## Abstract

**Objectives:**

To evaluate local tumor control and determinants of treatment success following magnetic resonance (MR)-guided microwave ablation (MWA) of liver malignancies.

**Materials and methods:**

This single-center retrospective study evaluated prospectively included patients undergoing MR-guided MWA between September 2021 and February 2025. Procedures were performed under general anesthesia using a 1.5-Tesla MR scanner and two MR-compatible MWA systems. Complications were assessed according to CIRSE. Technical success was defined as total lesion coverage on MR imaging one day post-ablation. Local recurrence and hepatic tumor progression were analyzed using follow-up liver MR imaging every three months. Determinants of treatment success included lesion size, tumor entity, ablation duration, MWA system, lesion and antenna conspicuity (6-point Likert scale), adjacent vascular structures, liver cirrhosis, and antenna position (central/non-central).

**Results:**

Forty-eight liver lesions in 40 patients (median age 62.5 years, 70.0% male) were treated. MWA systems 1 and 2 were used in 33/48 (68.8%) and 15/48 lesions (31.3%), respectively. Technical success was achieved in 46/48 lesions (95.8%), with one peri-procedural complication (CIRSE grade 3, 2.5%). Mean follow-up was 21.0 ± 10.9 months, with one patient lost to follow-up. Local recurrence occurred in 4/47 lesions (8.5%), all treated with system 1, while no recurrence was observed with system 2 (*p* = 0.173). As the only relevant determinant of treatment success, adjacent vascular structures were associated with higher local recurrence rates (*p* = 0.019). No local recurrence occurred in lesions < 20 mm.

**Conclusion:**

MR-guided MWA of liver tumors is safe, achieving high technical success and local tumor control. Adjacent vascular structures increase the risk of local recurrence and may warrant more aggressive treatment.

**Key Points:**

***Question**** Whether MR-guided MWA provides reliable local tumor control for liver malignancies and which procedural or anatomical factors influence treatment success*.

***Findings**** MR-guided MWA achieved high technical success, low complication rates, and strong local tumor control; local recurrence occurred in lesions ≥ 20 mm and with low-power MWA systems, as well as adjacent vascular structures, significantly increased risk of local recurrence*.

***Clinical relevance**** Prospectively acquired single-center cohorts of MR-guided MWA with real-time position monitoring in a standardized MR workflow, highlighting the impact of technical factors and the importance of lesion selection based on device-specific ablation characteristics*.

**Graphical Abstract:**

**Figure:**
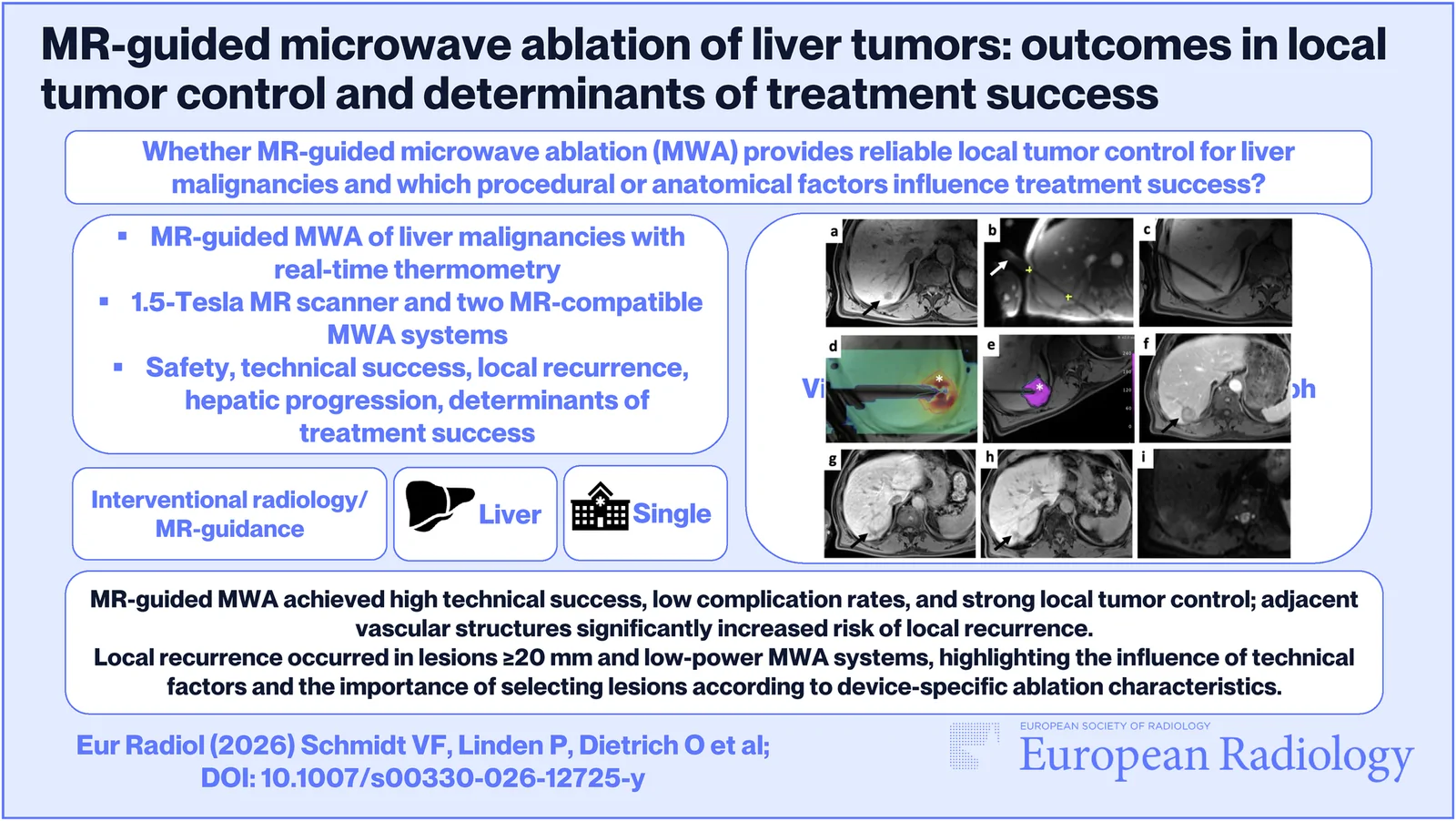

## Introduction

Minimally invasive thermal ablation has become an increasingly important treatment modality for primary and secondary liver malignancies, offering a potentially curative treatment alternative to surgical resection [1, 2]. Among available techniques, microwave ablation (MWA) has gained popularity due to its ability to achieve higher intratumoral temperatures, larger ablation volumes, and shorter procedural times compared to radiofrequency ablation (RFA) [3, 4]. Successful ablation, however, relies on precise targeting and benefits from real-time monitoring to ensure complete coverage of the lesion while preserving adjacent healthy liver tissue and critical vascular structures [5, 6]. Magnetic resonance (MR) imaging offers unique advantages for guiding liver interventions [7, 8]. Its superior soft tissue contrast facilitates accurate lesion localization and trajectory planning. Moreover, recent technological advancements—such as novel MR-compatible MWA systems, optimized interactive guidance software, and high-speed imaging sequences—allow increasingly precise and multiplanar verification of antenna placement [7]. In addition, MR thermometry allows continuous monitoring of tissue temperatures during ablation, providing critical feedback to optimize energy delivery and enhance procedural safety [9, 10]. Early reports suggest high rates of technical success and favorable local tumor control [11, 12]. Nevertheless, evidence regarding factors influencing procedural outcomes, such as lesion characteristics, adjacent vascular structures, the used MWA system, antenna positioning, and peri-procedural imaging parameters, remains limited. This study aimed to both evaluate outcomes in local tumor control after 6-, 12-, and 18 months, and to identify determinants of treatment success following MR-guided MWA of liver lesions.

## Materials and methods

### Study design

All patients undergoing MR-guided MWA of histopathologically proven liver malignancies with MR thermometry acquisition during 09/2021 and 02/2025 were included. Treatment decisions for MR-guided MWA were based on a multidisciplinary tumor board. This analysis was approved by the local ethics committee (protocol number 22-0227, 01/31/2024), and informed consent was waived because of the retrospective nature of the study. The patients included gave informed consent for the procedure and were recruited prospectively to a trial on sequence and workflow evaluation of the interventional MRI software package of the vendor after written informed consent. Details on MR-guided MWA, safety, and imaging protocols are presented in the Supplement.

### Determinants of treatment success

With local tumor control as the primary outcome measure, the influence of the following clinical and technical parameters was analyzed: lesion size, tumor entity, ablation duration, type of MWA system, lesion conspicuity, antenna visualization, adjacency to vessels, and antenna tip position with the target. Peri-interventional imaging parameters were evaluated by one reader with 10 years of experience in diagnostic liver MRI. The conspicuity of liver lesions and microwave antennas on peri-interventionally acquired T1-weighted VIBE and fluoroscopic BEAT interactive sequences was rated using a 6-point Likert scale (0 = not visible, 1 = poor, 2 = moderate, 3 = good, 4 = very good, 5 = excellent [sharply delineated]). Presence of relevant adjacent vascular structures (defined as hepatic and/or portal veins > 3 mm in diameter within 5 mm of the lesion; yes/no) or liver cirrhosis (yes/no), as well as the position of the antenna tip (central/non-central), was assessed based on peri-interventional T1-weighted VIBE images.

### Statistical analysis

Descriptive statistics were used to analyze the distribution of patients among the different categories. The Shapiro–Wilk test was used for the assessment of normality. Data are presented as means (± standard deviation) in case of normal distribution or as medians (interquartile range, IQR) for skewed distribution. To compare the groups with and without local recurrence, the Wilcoxon rank sum test, Pearson’s chi-squared test, and Fisher’s exact test were used. Statistical analysis was performed by using SPSS 31.0 statistical software (IBM SPSS Statistics). *p* < 0.05 was considered significant.

## Results

### Patient characteristics

The analysis included 40 patients with a total of 48 liver lesions treated with MR-guided MWA.

Of all patients, the mean body mass index (BMI) was 28.4 ± 4.3, and 28 patients (70.0%) were male. The median age at the time of treatment was 62.5 years (IQR, 57.0–68.0 years). Most patients had received prior treatment for the underlying malignancy (26/40, 65.0%), including systemic therapy (16/40, 40.0%), liver resection (10/40, 25.0%), transarterial chemoembolization (TACE; 6/40, 15.0%), and local ablative therapies (8/40, 20.0%). The tumor entities were hepatocellular carcinoma (HCC) in 18/40 (45.0%), liver metastases of colorectal carcinoma (CRLM) in 10/40 (25.0%), and of neuroendocrine tumors (NET) in 7/40 patients (17.5%). Additionally, liver metastases of uveal melanoma, breast carcinoma, and cholangiocellular carcinoma (CCC) were present in 2/40 (5.0%), 2 /40 (5.0%), and 1/40 patients (2.5%), respectively.

One lesion was treated in 32/40 patients (80.0%), while two lesions were treated in 8/40 patients (20.0%). Details on lesion location and size are provided in Table 1.

**Table 1:** Clinical characteristics of liver lesions

	Number	Median (IQR)
Liver malignancy	48 (100%)	
Hepatocellular carcinoma	22 (45.8%)	
Colorectal carcinoma	13 (27.1%)	
NET	8 (16.7%)	
Uveal melanoma	2 (4.2%)	
Breast carcinoma	2 (4.2%)	
CCC	1 (2.1%)	
Lesion location	48 (100%)	
Segment 1	0 (0.0%)	
Segment 2	4 (8.3%)	
Segment 3	2 (4.2%)	
Segment 4a	7 (14.6%)	
Segment 4b	1 (2.1%)	
Segment 5	3 (6.3%)	
Segment 6	5 (10.4%)	
Segment 7	10 (20.8%)	
Segment 8	16 (33.3%)	
Number of lesions ablated per session	40 (100%)	
1	32/40 (80.0%)	
2	8/40 (20.0%)	
Lesion size in mm		15.5 9.25–19.75

### Procedural details and safety

**Fig. 1:**
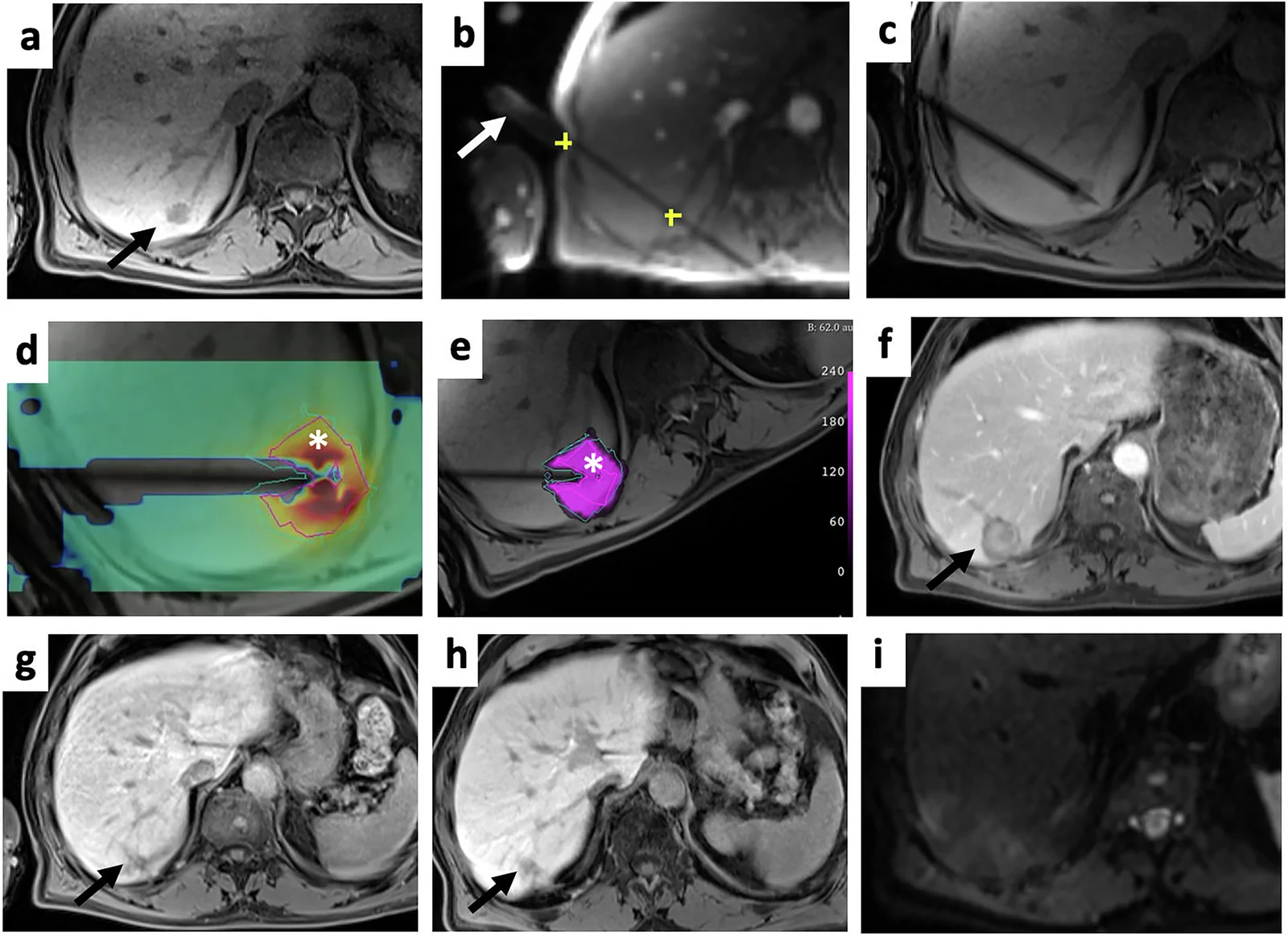
Eighty-year-old male with metastatic colorectal carcinoma undergoing MR-guided MWA using the ECO-200G system. **a** Axial T1-weighted (T1w) MRI after injection of gadoxetic acid shows a liver lesion (arrow) in segment 7. **b** Defining and digital marking of entry point and target lesion (yellow tags) for procedure planning in use of real-time T1w MR sequences and finger-pointing (arrow). **c** Periprocedural axial T1w MRI confirmed optimal antenna placement. **d** MRI thermometry image shows temperature distribution within the liver during the ablation (asterisk). **e** Thermal dose image shows the predicted volume exposed to a lethal dose of thermal energy (asterisk). **f** Portal-phase MRI on the first postprocedural day shows the extent of the ablation zone (arrow), complete coverage of the lesion was achieved. **g**–**i** Axial T1w MR images in the hepatobiliary phase after three (**g**) and six (**h**) months, as well as diffusion-weighted imaging (DWI) after six months (**i**), reveal a decrease in lesion size accompanied by corresponding post-therapeutic changes consistent with complete response (arrows)

**Fig. 2:**
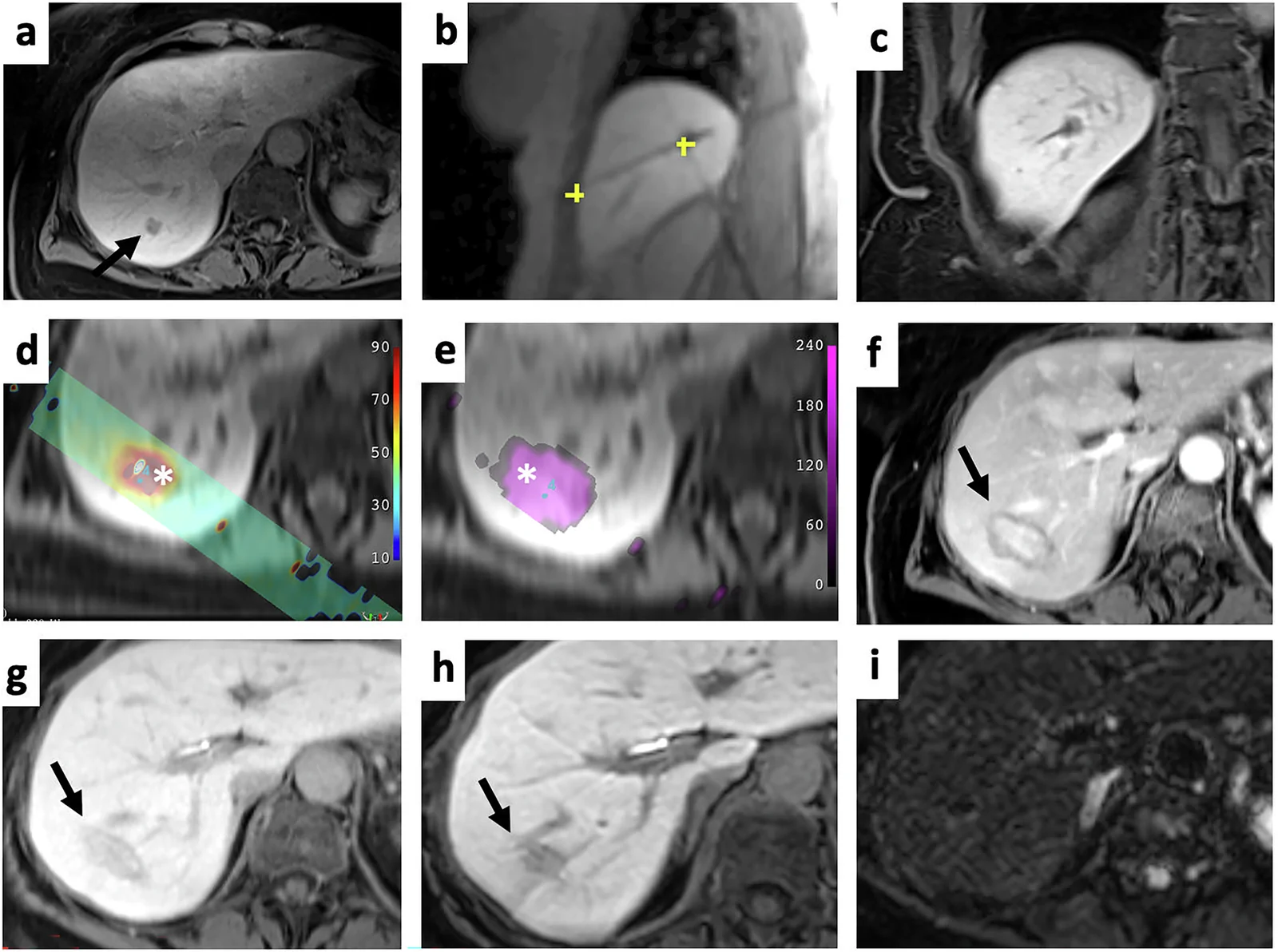
Sixty-six-old female with metastatic uveal melanoma undergoing MR-guided MWA using the AveCure system. **a** Axial T1-weighted (T1w) MRI after injection of gadoxetic acid shows a liver lesion (arrow) in segment 7. **b** Defining and digital marking of entry point and target lesion (yellow tags) for procedure planning in use of real-time T1w MR sequences and finger-pointing. **c** Periprocedural coronal T1w MRI confirmed optimal antenna placement. **d** MRI thermometry image shows temperature distribution within the liver during the ablation (asterisk). **e** The thermal dose image shows the predicted volume exposed to a lethal dose of thermal energy (asterisk). **f** Portal-phase MR image on the first postprocedural day shows the extent of the ablation zone (arrow); complete coverage of the lesion was achieved. **g**–**i** Axial T1w MR images in the hepatobiliary phase after three (**g**) and six (**h**) months, as well as diffusion-weighted imaging (DWI) after six months (**i**), reveal a decrease in lesion size accompanied by corresponding post-therapeutic changes consistent with complete response (arrows)

Of the 48 lesions, 33/48 (68.8%) were treated with system 1 (AveCure, MedWaves), 15/48 (31.3%) using system 2 (ECO-200G, ECO Medical Instruments Co., Ltd.), see Figs. 1 and 2. Delivered energy for the system 1 was 12.5 kJ (IQR, 10.0–17.8), while the applied power for the system 2 was 100 W (IQR, 100–115); the overall ablation duration (*n* = 48) was 10.5 min (IQR, 8.25–12.0), with 10.0 min (IQR, 8.5–12.0) for system 1 and 11.0 min (IQR, 8.0–15.0) for system 2. Further details are provided in Table 2.

**Table 2:** Procedural details of MR-guided MWA

	Number	Median (IQR)
Liver lesions	48 (100%)	
Used MWA system		
AveCure	33 (68.8%)	
ECO-200G	15 (31.3%)	
Delivered energy (AveCure) in kJ		12.5 (10–17.8)
Applied power (ECO-200G) in W		100 (100–115)
Ablation duration in min (*n* = 48)		10.5 (8.25–12.0)
AveCure		10.0 (8.5–12.0)
ECO-200G		11.0 (8.0–15.0)
Antenna repositioning during ablation	8/48 (16.7%)	
Delivered energy (AveCure) in kJ after repositioning (*n* = 6)		14 (7.75–19.75)
Applied power (ECO-200G) in W after repositioning (*n* = 2)		100 (100–100)
Ablation duration in min (*n* = 8)		10 (3–11.5)
AveCure		10 (8.25–11.75)
ECO-200G		3 (not applicable)^+^

*kJ* kilojoule, *min* minutes, *W* watts

^+^ Sample size too small; AveCure: MedWaves; ECO-200G: ECO Medical Instruments Co.

Peri- and postprocedural complications occurred in one procedure (1/40, 2.5%). In this case, a progressive pleural effusion was noted on the MRI control sequence at the end of MWA. Due to clinical suspicion of bleeding, a digital subtraction angiography (DSA) was performed, which revealed no active bleeding. The patient remained stable after the DSA, with no sequelae remaining (CIRSE grade 3).

### Technical success and tumor control rates

Postprocedural MR imaging performed one day after MWA demonstrated complete coverage (technical success) in 46/48 lesions (95.8%), whereas 2/48 lesions (4.2%) were incompletely covered by the ablation zone (technical failure). In both cases, insufficient safety margins ≤ 5 mm were located adjacent to larger portal venous vessels.

The mean maximum follow-up period was 21.0 ± 10.8 months. One of 40 patients (2.5%) was lost to follow-up, resulting in 47/48 lesions (97.9%) with follow-up. Local recurrence of the ablated lesions was observed in 4/47 lesions (8.5%), with a mean time to local tumor progression (TLTP) of 8.9 ± 7.1 months. Hepatic tumor progression (outside the ablation zone) was observed in 17/39 patients (43.6%), with a mean time to non-target progression (TNTP) of 10.3 ± 7.5 months.

Local recurrence-free survival (LRFS) rates were 94.1%, 93.3%, and 91.3% at 6, 12, and 18 months, respectively. Hepatic progression-free survival (HPFS) and progression-free survival (PFS) rates were 88.2% and 82.4% at 6 months, 73.3% and 66.7% at 12 months, and 60.9% and 52.2% at 18 months, respectively. In all six cases with technical failure or local recurrence, repeat local ablative procedures were performed in each patient (6/6, 100%). Of the 17 cases with hepatic tumor progression, systemic therapy was adjusted in 10/17 patients (41.5%), repeat local ablative procedures were performed in 6/17 cases (35.3%), and TACE and radioembolization were performed in one case each (5.9%).

### Determinants of treatment success

Lesion size differed numerically between local recurrence and no local recurrence groups but did not reach statistical significance (20.5 mm vs 14 mm; Wilcoxon rank sum test, *p* = 0.107). Tumor entity and ablation duration showed no significant influence on local recurrence (Fisher’s exact test, *p* = 0.712; Wilcoxon rank sum test, *p* = 0.107; Table 3). All cases of local recurrence were treated with system 1 (4/4, 100%), whereas no cases of progression occurred with system 2; however, this difference was not statistically significant (Fisher’s exact test, *p* = 0.173). There was no relevant difference in lesion size between the two systems (system 1, median 16 mm, IQR 10–20 mm vs system 2, median 13 mm, IQR 8–19 mm; *p* = 0.213). No local recurrence occurred in lesions < 20 mm.

**Table 3:** Clinical/technical characteristics and determinants of treatment success

	Local tumor recurrence (*n* = 4)^*^	No local tumor recurrence (*n* = 43)^*^	*p* value
Lesion size in mm; median (IQR)	20.5 (20–30.75)	14 (9–18)	0.107^#^
Tumor entity			0.712^+^
HCC	3/4 (75.0%)	18/43 (43.9%)	
Colorectal carcinoma	1/4 (25.0%)	12/43 (26.8%)	
NET	0/4 (0.0%)	8/43 (19.5%)	
Uveal melanoma	0/4 (0.0%)	2/43 (4.9%)	
Breast carcinoma	0/4 (0.0%)	2/43 (2.4%)	
CCC	0/4 (0.0%)	1/43 (2.4%)	
Ablation duration in min; median (IQR)	13 (12–14.75)	10 (7.75–12)	0.107^#^
Type of MWA system			0.173^+^
AveCure	4/4 (100%)	29/43 (70.7%)	
ECO-200G	0/4 (0.0%)	14/43 (29.3%)	
Median VIBE lesion conspicuity; median (IQR)	3.5 (1.5–4.0)	4.0 (3.0–4.0)	0.765^#^
Median VIBE antenna conspicuity; median (IQR)	4.0 (4.0–4.75)	5.0 (4.0–5.0)	Not applicable^##^
Median *BEAT* lesion conspicuity; median (IQR)	3.5 (3.0–4.0)	4.0 (3.0–4.0)	0.601^#^
Median *BEAT* antenna conspicuity; median (IQR)	4.5 (4.0–5.0)	4.0 (4.0–5.0)	0.802^#^
Adjacent vascular structures			0.019^+^
Yes	3/4 (75.0%)	6/43 (14.6%)	
No	1/4 (25.0%)	37/43 (85.4%)	
Liver cirrhosis			0.610
Yes	2/4 (50.0%)	19/43	
No	2/4 (50.0%)	24/43	
Antenna tip position			1.000^+^
Central	1/4 (25.0%)	13/43 (31.7%)	
Non-central	3/4 (75.0%)	30/43 (68.3%)	

*BEAT* BEAT interactive MRI sequence (Siemens Healthineers), *VIBE* Volumetric interpolated breath-hold examination magnetic-resonance sequence

^*^ *n* = 47, as one patient with one treated lesion was lost to follow-up

^#^ Wilcoxon rank sum test

^##^ All test field values were ≤ the median

^+^ Fisher’s exact test

The median VIBE (volumetric interpolated breath-hold examination MRI sequence) lesion conspicuity was rated 4 (IQR 3–4), and the median VIBE antenna conspicuity was 5 (IQR 4–5). The median *BEAT* (interactive MRI sequence, Siemens Healthineers) lesion conspicuity was 4.0 (IQR 3–4), while the median *BEAT* antenna conspicuity was 4 (IQR 4–5). Relevant adjacent vascular structures were found in 10/48 lesions (20.8%). A non-central position of the antenna tip relative to the lesion was observed in 14/48 lesions (29.2%), respectively.

The evaluated lesion and antenna conspicuity showed no significant influence on local recurrence (Wilcoxon rank sum test, *p* = 0.765, *p* = 0.601, and *p* = 0.802). In contrast, the presence of adjacent vascular structures had a significant impact on local recurrence (Fisher’s exact test: *p* = 0.019). The position of the antenna tip, however, was not identified as a significant predictor (Fisher’s exact test: *p* = 1.000). In non-central antenna positions, the lesion size was significantly smaller with a median of 9 mm (IQR 8–11.25 mm), compared to central antenna positions (17 mm, IQR 13–20 mm; Wilcoxon rank sum test, *p* < 0.002). All detailed results are summarized in Table 3.

## Discussion

This study demonstrates that MR-guided MWA provides high technical success, low complication rates, and excellent local tumor control, with low recurrence rates over mid-term follow-up. While no significant predictors of local recurrence were identified among lesion or procedural factors, the presence of adjacent vascular structures emerged as the only relevant determinant. Notably, all local recurrences occurred with the AveCure system (system 1), whereas no local recurrences were observed with the ECO-200G system (system 2). Interventional MRI remains an evolving field, and this study represents one of the largest prospectively acquired single-center cohorts of MR-guided MWA combined with real-time position monitoring within a standardized clinical MR workflow to date.

The high technical success rate of > 95% is consistent with the broader literature on MWA of liver tumors (73.1–100%) [13, 14]. In a multinational registry evaluation of 136 centers, complete ablation was achieved in 93.3% of 4703 liver lesions treated by CT-guided MWA [15]. Various strategies have been described to maximize the technical success rate regarding achieving complete ablation. Under CT guidance, these range from anatomical landmark–based orientation to intraprocedural contrast administration [16, 17]. A multicenter CT-guided MWA study reported a complete ablation rate of 99.4% using intraprocedural contrast-enhanced CT for margin assessment and additional ablations [16]. However, real-time target visualization remains a challenge. For MR-guided MWA, very high technical success rates have consistently been reported [18–21], likely due to superior lesion visualization. Winkelmann et al [22], Weiss et al [23], and Chen et al [24] reported 100% technical success in cohorts of 28, 58, and 74 liver lesions, with mean target sizes of 15, 16, and 13 mm, respectively. This is supported by a review encompassing five studies that reported a complete ablation rate of 98.8% in a total of 75 patients [25]. Cases of incomplete MR-guided ablation may be most likely attributable to big tumor sizes or relevant adjacent vascular structures with consecutive heat-sink effect [26]. In the present study, the insufficient safety margin < 5 mm in both incomplete cases was adjacent to larger portal venous vessels. The favorable complication rate of 2.5% is lower than or comparable to published literature [23, 24, 27]. Weiss et al reported minor and major complication rates of 20% and 6% [23], whereas Liu et al described complications in 7.7% [27]. Differences between CT- and MR-guided MWA [28] may relate to improved visualization of critical structures under MR guidance, particularly with hepatocyte-specific contrast agents [7], although other studies found no significant difference [29]. Importantly, the single grade-3 complication highlights that higher-grade events may occur, requiring careful peri- and post-procedural monitoring. The local recurrence rate of 8.5%, with a mean TLTP of 8.9 months, is comparable to MR-guided cohorts (1.9–15.8%) [18, 21, 23, 24]. Similar or higher local recurrence rates have been reported for CT-guided MWA [3, 13, 30], with a large review showing local recurrence rates of 5–17.8% for HCC and 10–35.7% for liver metastases [3]. Although described less systematically and less frequently, reported LRFS, hepatic tumor progression rates, HPFS, and PFS are comparable to those observed in the present study and represent the natural course of disease at a similar patient selection [14]. Weiss et al reported a hepatic tumor progression rate of 64% after a median TNTP of 13.8 months [23], compared to 43.6% after 10.3 months in our cohort. Among the investigated factors, adjacent vascular structures emerged as the most important determinant of local tumor control. This is attributable to convective heat loss caused by blood flow, which counteracts the ablative effect. The thermal sink effect is particularly relevant when ablation depends on the passive heating zone adjacent to vessels, where reduced energy deposition fails to achieve cytotoxic temperatures in perivascular tissue [3]. It is substantially more pronounced in RFA, which depends on electrically mediated resistive heating and is limited by tissue conductivity and impedance, whereas MWA generates heat via electromagnetic fields, allowing higher and more consistent energy delivery [3]. Nevertheless, our findings demonstrate that the thermal sink effect may also be clinically relevant during MWA and should be considered in the presence of large portal vein branches and high blood flow. Related to presently available MRI-compatible MWA systems, including several low microwave posers, these observations emphasize careful pre-procedural planning and, in selected cases, more aggressive ablation strategies, including antenna repositioning or prolonged ablation duration. These limitations, however, might be overcome with more powerful devices. In our cohort, lesion size, tumor entity, ablation duration, antenna and lesion conspicuity, presence of liver cirrhosis, and antenna position showed no significant influence on local tumor control. Some studies identified lesion size as a significant determinant—with an increased risk of local recurrence typically observed only for lesions larger than 20 or 30 mm [31–33]; this may not apply here due to the generally small and relatively uniform lesions (median 15.5 mm, IQR 9.25-19.75). This size homogeneity may also reflect a selection bias related to MR-guided therapy and should be considered when interpreting the favorable local recurrence rate. However, we observed a trend towards a higher local recurrence rate in larger lesions (median 20.5 vs 14 mm). Regarding tumor entities, the literature reports heterogeneous findings: some studies have described higher local recurrence rates after MWA for metastases (e.g., CRLM) compared to HCC [33, 34], while others report no significant differences, particularly for small lesions < 5 mm [13, 35]. In our cohort, three recurrences occurred in HCC lesions; however, these were mostly associated with adjacent vascular structures, and HCC represented the largest subgroup (45.8%). In this study, both lesion and antenna conspicuity were generally high with little variability and had no significant impact on treatment success or local tumor control. Likewise, non-central antenna positioning was not a significant determinant, likely because it occurred mainly in very small lesions (median size of 9 mm), where central placement is technically challenging; complete ablation with adequate minimal margins was still achieved.

All cases of incomplete ablation and local recurrence occurred with the AveCure system (system 1), while none were observed with the ECO‑200 G (system 2), though this difference was not statistically significant due to the small sample size. These findings should not be interpreted as a direct comparison, as they likely reflect technical differences rather than true performance: AveCure operates at a lower frequency (902–928 MHz) and output power (40 W), using temperature–controlled energy delivery and no active antenna cooling [36]. In contrast, ECO‑200 G operates at higher frequencies (2.45 GHz) and output power (100 W) with water‑cooled antennas, enabling larger ablation zones and more stable energy delivery [37]. AveCure, however, allows simpler hardware and direct in-room use within the MR scanner. The interventional radiologist’s learning curve may also have influenced results, as AveCure was used earlier and ECO-200G later. Notably, no recurrences occurred in small lesions (< 20 mm), including those treated with system 1. This study has several limitations: First, its retrospective design, although based on a prospectively maintained in-house database established as part of a trial on sequence optimization. Second, the small sample size limits statistical power, particularly for more granular subgroup analyses, such as comparisons between different MWA systems. Third, the heterogeneity of the cohort, comprising multiple tumor entities, may introduce variability and limit the generalizability of entity-specific conclusions; however, pan-cancer oligometastatic treatment concepts are increasingly being adopted. MR-guided ablation was preferentially performed for smaller lesions, introducing potential selection bias that may have influenced local tumor control outcomes. Another potential bias relates to the sequential implementation of the two MWA systems, with system 1 used initially and system 2 introduced later. This temporal separation may have introduced confounding through increasing operator experience over time. Further validation is needed, and these results form the basis for an ongoing prospective study (MR-MIGHTY) investigating local tumor control after MR-guided MWA with a target rate of 96%.

## Conclusion

MR-guided MWA of liver tumors is safe, achieving high technical success and favorable local tumor control. Relevant adjacent vascular structures should be carefully identified, as their presence increases the risk of local recurrence and may warrant more aggressive treatment strategies. All cases of local recurrence occurred in lesions ≥ 20 mm and with a low-power MWA system, underscoring the potential impact of technical factors and the need for careful selection of lesions according to the specific technical characteristics of the device.

## Supplementary information

ELECTRONIC SUPPLEMENTARY MATERIAL

## Abbreviations

BEAT: Interactive MRI sequence
CCC: Cholangiocellular carcinoma
CRLM: Liver metastases of colorectal carcinoma
DSA: Digital subtraction angiography
HCC: Hepatocellular carcinoma
HPFS: Hepatic progression-free survival
IQR: Interquartile range
LRFS: Local recurrence-free survival
MWA: Microwave ablation
NET: Neuroendocrine tumor
PFS: Progression-free survival
RFA: Radiofrequency ablation
TACE: Transarterial chemoembolization
TLTP: Time to local tumor progression
TNTP: Time to non-target progression
VIBE: Volumetric interpolated breath-hold examination MRI sequence
