# Supplementary material for: MR-guided microwave ablation of liver tumors: outcomes in local tumor control and determinants of treatment success
Source: Eur Radiol. 2026 Jul 4;36(11):8052–61. doi: 10.1007/s00330-026-12725-y (PMC13661726; doi:10.1007/s00330-026-12725-y)
Supplement: Supplementary file 1 [file 330_2026_12725_MOESM1_ESM.pdf]

MR-Guided Microwave Ablation of Liver Tumors: Outcomes in Local Tumor Control and Determinants of Treatment Success

ELECTRONIC SUPPLEMENTARY MATERIAL

Eur Radiol (2026) Schmidt VF; Linden P; Dietrich O et al.

Baseline imaging

In accordance with the standard of care, all patients underwent oncologic staging prior to the procedure, including contrast-enhanced (Iomeron 300, Bracco Ltd., Milano, Italy) CT as well as liver MRI including a hepatobiliary phase acquired 10 minutes after the injection of gadoxetate disodium (Primovist®, Bayer Vital, Leverkusen, Germany).

MR-guided MWA

All MWA procedures were performed under general anesthesia by a single senior interventional radiologist with over 20 years of experience in image-guided interventions. Procedures were conducted using a closed-bore whole-body 1.5-tesla (T) MRI system (MAGNETOM Aera, subsequently upgraded to MAGNETOM SolaFit; Siemens Healthineers, Forchheim, Germany), featuring a short-bore design (cover-to-cover system length 145 cm, bore diameter 70 cm), a maximum gradient strength of 45 mT/m, and a slew rate of 200 T/m/s. Signal reception was achieved using a 110-mm receive-only surface loop coil in combination with the built-in 12-element spine array, allowing an open anterior approach to the liver. An MRI-compatible in-room liquid crystal display monitor (InroomViewingDevice LCD 3.1; NordicNeuroLab, Bergen, Norway) was installed adjacent to the scanner for real-time procedural monitoring/guidance.

MWA was performed using MR-conditional microwave ablation systems (system 1: AveCure, MedWaves, San Diego, CA, USA, or system 2: ECO-200G, ECO Medical Instruments Co., Ltd., Nanjing, China) equipped with a 14- or 15-gauge microwave antenna (system 1 and 2) featuring a 1–4-mm active tip. With both systems, single-antenna ablations were performed in continuous-ablation mode. MR-compatible coaxial extension cable lengths were 7.2 m (system 1) and 5.0 m (system 2).

Approximately 10 minutes after intravenous administration of a weight-based dose (0.1 mL/kg body weight) of a hepatospecific contrast agent (gadoxetate disodium; Primovist, Bayer Vital, Leverkusen, Germany), axial and coronal T1-weighted Dixon gradient-echo (GRE) sequences were acquired for lesion localization (flip angle 10°, repetition time [TR] 6.8 ms, echo times [TE] 2.4 and 4.8 ms, slice thickness 3 mm, matrix 320 × 195, bandwidth 470 Hz/pixel, field of view 380 × 380 mm², breath-hold acquisition, reconstruction of water images). The access trajectory was planned using the Needle Intervention Add-In (Siemens Healthineers). After defining the skin entry point by laser and subsequent finger pointing, sterile draping and local anesthesia were applied. Free-hand antenna placement was performed under real-time fluoroscopic T1-weighted GRE guidance using the *BEAT interactive* sequence (Siemens Healthineers), enabling sequential imaging in three orthogonal planes (flip angle 30°, TR 7.5 ms, TE 3.6 ms, slice thickness 5 mm, field of view 380 × 380 mm², matrix 128 × 128, GRAPPA acceleration R=2, bandwidth 250 Hz/pixel, no phase oversampling, no breath-hold or respiration compensation trigger, acquisition time: 0.57 s/frame). Correct antenna positioning within the target lesion was subsequently confirmed on two orthogonal diagnostic T1-weighted Dixon GRE images. Depending on lesion size and the ablation system used, power settings and emission duration or target temperature and emission time were selected in accordance with the manufacturer's recommendations. If the antenna placement was considered inadequate during the procedure, it was adjusted and the ablation was continued or repeated as needed. During ablation, proton resonance frequency (PRF)–based MR thermometry was then performed using phase images acquired with a gradient-echo single-shot echo-planar imaging (EPI) sequence (C2P research sequence; 13 slices, slice thickness 3 mm, in-plane resolution 2.8 × 2.8 mm², field of view 360 × 360 mm²) to enable periprocedural prediction of the ablation zone, as reported previously (1). No

protective techniques (e.g., hydrodissection or carbodissection) were used during ablation. Track ablation was performed in each procedure. Axial T2w fat-saturated turbo spin echo and T1w GRE sequences were obtained at the end of each treatment.

Safety

All patients underwent MWA as an in-house procedure with one night observation. Coagulation status (thrombocytes >50.000/µl, INR <1.5) and absence of contraindications to enhanced MRI had been screened standardized before the procedures. During postprocedural hospitalization all patients were observed for potential complications including close electronical vital sign monitoring (blood pressure, heart rat, oxygen saturation) for 2h in anesthesia recovery room. In parallel, abdominal US imaging was routinely performed to exclude possible hematoperitoneum. For detection of possible subclinical bleeding, hematocrit levels were checked 4h postprocedural. All signs of peri- or postprocedural complications were classified as major and minor according to the CIRSE classification (2).

Postprocedural imaging of lesion coverage and tumor control

To evaluate technical success/failure, lesion coverage (total/incomplete) through the ablation zone was assessed in consecutive MR images on a 1.5 T MR system (MAGNETOM Avanto, MAGNETOM Aera, Siemens Healthineers, Forchheim, Germany) one day after the procedure, as the ablation zone is known to continue evolving during the first hours following treatment (3). Complete lesion coverage was defined as lesion coverage with a consistent safety margin >5 mm. The MRI protocol included T2w axial HASTE and T1w GRE sequences with fat suppression (volumetric interpolated breath-hold examination; VIBE) before and 20, 50, and 120 s after

intravenous injection of 0.1 ml/kg Gadobutrol (Bayer Vital, Leverkusen, Germany; 0.1 mmol/kg body weight). Local recurrence (yes/no) and hepatic progression (yes/no) was analyzed using diagnostic CT (every 6 months) and liver MRI (every 3 months) performed at consecutive routine follow-up in our out-patient clinic which included a hepatobiliary phase (10 min after the injection of gadoxetate disodium; Primovist, Bayer Vital, Leverkusen, Germany). Local recurrence–free survival (LRFS) and hepatic progression–free survival (HPFS) rates were calculated at 6, 12, and 18 months. LRFS was defined as time to local tumor recurrence, HPFS as time from ablation to intrahepatic progression or death from any cause. Progression-free survival (PFS) was defined as time from ablation to local progression, new intrahepatic or extrahepatic disease, or death from any cause.

References

1. Öcal O, Dietrich O, Lentini S, et al. (2024) Predicting liver ablation volumes with real-time MRI thermometry. JHEP Rep. 10.1016/j.jhepr.2024.101199
2. Filippiadis DK, Binkert C, Pellerin O, et al. (2017) Cirse Quality Assurance Document and Standards for Classification of Complications: The Cirse Classification System. Cardiovasc Intervent Radiol. 10.1007/s00270-017-1703-4
3. Ringe KI, Wacker F, Raatschen HJ (2015) Is there a need for MRI within 24 hours after CT-guided percutaneous thermoablation of the liver? Acta Radiol. 10.1177/0284185114520858
